# Prevalence and Risk Factors Associated with Tumors and Other Structural Anomalies in Brain MRI Performed to Rule out Secondary Headache: A Multicenter Observational Study

**DOI:** 10.3390/ijerph19063521

**Published:** 2022-03-16

**Authors:** José Pablo Martínez Barbero, Antonio Jesús Láinez Ramos-Bossini, Mario Rivera-Izquierdo, Francisco Sendra-Portero, José Manuel Benítez-Sánchez, Jorge A. Cervilla

**Affiliations:** 1 Department of Radiology. Hospital Universitario Virgen de las Nieves, 18014 Granada, Spain; jpmbhg@hotmail.com; 2 Instituto de Investigación Biosanitaria de Granada (ibs.GRANADA), 18014 Granada, Spain; mariorivera@ugr.es (M.R.-I.); jcervilla@ugr.es (J.A.C.); 3 Department of Psychiatry, University of Granada, 18014 Granada, Spain; 4 Department of Preventive Medicine and Public Health, Hospital Universitario San Cecilio, 18016 Granada, Spain; 5 Department of Radiology and Physical Medicine, University of Malaga, 29071 Malaga, Spain; sendra@uma.es; 6 Department of Computer Science and Artificial Intelligence, University of Granada, 18071 Granada, Spain; j.m.benitez@decsai.ugr.es

**Keywords:** risk factors, prevention, neoplasms, headache, magnetic resonance imaging, central nervous system

## Abstract

Headache disorders (HDs) are among the most common conditions of the central nervous system, with an estimated prevalence of 50% in adult population. The aim of this work is to analyze the prevalence of structural anomalies that may explain HDs in MRI exams performed to rule out secondary headache in real-world practice, as well as risk factors associated with these lesions. We conducted a retrospective observational study based on a consecutive case series of all patients that underwent brain MRI due to headache from 1 January 2019 to 31 May 2019. We included patients from six MRI diagnostic centers accounting for four provinces of Andalusia (southern Spain). Bivariate and multivariate logistical regression models were performed to identify risk factors associated with the outcomes (1) presence of a structural finding potentially explaining headache, (2) presence of intracranial space-occupying lesions (SOLs), and (3) presence of intracranial tumors (ITs). Of the analyzed sample (1041 patients), a structural finding that could explain headache was found in 224 (21.5%) patients. SOLs were found in 50 (6.8%) patients and ITs in 12 (1.5%) patients. The main factors associated with structural abnormalities were female sex (OR, 1.35; 95% CI, 1.02–1.85), accompanying symptoms (OR, 1.34; 95% CI, 1.05–1.89), use of gadolinium-based contrast agents (OR, 1.89; 95% CI, 1.31–2.72) and previously known conditions potentially explaining headache (OR, 2.44; 95% CI, 1.55–3.84). Female sex (*p* = 0.048) and accompanying symptoms (*p* = 0.033) were also associated with ITs in bivariate analyses. Our results may be relevant for different medical specialists involved in the diagnosis, management and prevention of headache. Moreover, the risk factors identified in our study might help the development of public health strategies aimed at early diagnosis of brain tumors. Future studies are warranted to corroborate our findings.

## 1. Introduction

Headache disorders (HDs) are among the most common conditions of the central nervous system, with an estimated prevalence of 50% in the adult population [1]. They represent the leading cause of disability worldwide in people younger than 50 years (particularly in women), and a major cause of tremendous losses to the global economy [2]. In addition, it carries a high healthcare cost, as it is one of the most common reasons for medical consultation in emergency departments [3]. From an etiological perspective, HDs are classified as primary (absence of structural abnormalities and meeting specific diagnostic criteria) or secondary (with a defined and identifiable structural anomaly of diverse nature). However, this distinction is not always straightforward, and specific clinical guidelines have been published to help in doubtful cases. For example, according to the International Classification of Headache Disorders 3rd edition (ICHD-3), when a headache occurs for the first time in close temporal relation to another disorder known to cause headache, or fulfils other criteria for causation by that disorder, the new headache is coded as a secondary headache attributed to the causative disorder [4]. Of note, about 90% of HDs are of primary type [5], with migraine and tension-type headache being the most prevalent forms of primary headache [6], which makes them of special social and epidemiological interest [7].

Headache accounts for 18% of the overall medical reasons to order brain neuroimaging (i.e., computed tomography (CT) and magnetic resonance imaging (MRI)) studies [8]). International guidelines have been developed to provide appropriate indications for neuroimaging in HDs, including the NICE guidelines [9] and the American College of Radiology (ACR) appropriateness criteria [10]. The latter include different indications to perform neuroimaging examinations depending on the clinical scenario, with a degree of recommendation for each imaging modality (e.g., CT, MRI with or without intravenous contrast). Accordingly, patients with primary headache meeting diagnostic criteria should not undertake brain MRI if neurological examination is normal [10,11] since it does not add clinically-relevant information [12,13]. Nevertheless, in real-world practice, it is common that such a heterogeneous and prevalent condition does not fully meet the exact diagnostic criteria of specific clinical entities, being difficult for clinicians to label a HD as “primary”. 

Accordingly, neuroimaging examinations are commonly performed in clinical practice to rule out secondary causes of headache. From a diagnostic point of view, brain MRI and CT exams should be able to answer a clinical question posed by the ordering physician, namely identifying a specific condition that may explain patient’s symptoms [14]. However, this includes a myriad of possible diagnoses that are not always explicitly formulated, such as tumor, abscess, subdural hematoma, or stroke. Most of these structural abnormalities are included under the umbrella term ‘space-occupying lesions’, although other anomalies such as Chiari malformation can also cause a HD. In this setting, brain MRI is considered the exam of choice in the absence of recent trauma or acute onset, as it has shown higher sensitivity and specificity values than CT [15]. 

One of the most worrisome space-occupying lesions associated with headache are intracranial tumors (ITs). Overall, they represent an uncommon cause of headache (approximately 10% of secondary headaches) [16]. However, considering its clinical relevance and potential curability, they make up an essential focus of neuroimaging. Moreover, the influence of other variables (e.g., ordering specialist, imaging protocols used) might influence the positive predictive value of MRI and merits further analysis. Of note, so-called pseudo-false positive results—true abnormalities that will likely never be clinically relevant—can often lead to considerable distress for patients [17]. Therefore, comprehensive understanding of the prevalence and significance of structural abnormalities on brain MRI is needed to guarantee appropriate indications for this imaging examination.

The aim of this work is to analyze the prevalence of structural anomalies that may explain HDs in MRI exams performed to rule out secondary headache in real-world practice, as well as risk factors associated with these lesions.

## 2. Materials and Methods

We followed the Strengthening the Reporting of Observational studies in Epidemiology (STROBE) guidelines [18] for reporting the results of this study.

### 2.1. Study Design and Sample

We conducted a retrospective observational study based on a consecutive case series of all patients that underwent brain MRI due to headache from 1 January 2019 to 31 May 2019. We included patients from six MRI diagnostic centers (Algeciras, Cabra, El Ejido, Jaén, Jerez, and Zona Franca) accounting for four provinces of Andalusia (Almería, Cádiz, Córdoba, and Jaén). All MRIs undertaken in these centers were referred from a total of 11 public hospitals (Complejo Hospitalario Torrecárdenas, Complejo Hospitalario de Jaén, Hospital San Juan de la Cruz, Hospital de Poniente, Hospital Infanta Margarita, Hospital Universitario Puerta del Mar, Hospital Universitario de Puerto Real, Hospital de Jerez de la Frontera, Hospital Punta de Europa, Hospital Militar de San Carlos, U.S. Naval Hospital Rota) and private healthcare companies. Inclusion criteria were: patient’s age > 3 years; headache as main reason for MRI order, and imaging modality: contrast or non-contrast-enhanced brain MRI. Exclusion criteria included incomplete studies (claustrophobia, non-diagnostic quality MRI, equipment failure, etc.) and lack of outcome data (i.e., missing or incomplete radiology report). All studies were performed in 1.5 T MRI machines with similar technical parameters and imaging protocols, determined by the clinical scenario based on the ACR appropriateness criteria. 

### 2.2. Data Collection and Variables

The information collected through our radiology information system (RIS) was assessed by two independent researchers. Once the sample was obtained, MRIs were read by a neuroradiologist who was blinded to the initial report and patient outcomes. Discrepancies between both readers were solved by consensus with a third neuroradiologist. In addition, the medical records of the patients were consulted to collect relevant sociodemographic and clinical information. The variables considered for this study as exposure variables were age, sex, province (Almería, Cádiz, Córdoba, or Jaén), medical specialty that requested the MRI, type of headache, use of gadolinium-based contrast agents (GBCAs), history of headache previous to the current episode, presence of concomitant symptoms, trauma, previous brain MRI exams, and previously-known conditions that may justify headache (e.g., cancer). The outcome variables were (1) presence of a structural finding that could explain headache, (2) presence of intracranial space-occupying lesions, and (3) presence of intracranial neoplastic space-occupying lesions (i.e., ITs). 

### 2.3. Statistical Analyses

First, we conducted a univariate descriptive analysis. Absolute frequencies (count) and relative frequencies (percentage) were calculated for qualitative variables. Similarly, means and standard deviation (SD) were considered for quantitative variables (age). 

Second, we performed bivariate analyses to detect potential associations between the collected variables and each of the three outcomes of interest. For continuous variables, T-tests were applied. For qualitative variables, chi-square tests were used and, when the conditions for application were not met, Fisher’s exact tests were applied.

Finally, multivariate logistic regression models were performed to evaluate the independent adjusted effect of the variables detected in bivariate analyses with respect to outcome 1 (presence of a structural finding that could explain headache). Odds ratios (OR) were calculated with adjustments for age, sex, province, type of headache, use of GBCAs, concomitant symptoms, previous MRIs, and previously known conditions that may justify headache (e.g., cancer). No multivariate analyses were conducted on the other two outcome variables given the low number of patients in these groups. All statistical analyses were performed using R software version 4.0.3 (R Foundation for Statistical Computing, Vienna, Austria).

### 2.4. Ethical Considerations

This study was approved by the Provincial Ethics Committee of Granada (code: 2650-N-20). We used an anonymized database, and all potentially identifiable data were removed before analyses. This study complies with the ethical standards stated in the Declaration of Helsinki.

## 3. Results

**Figure 1:**
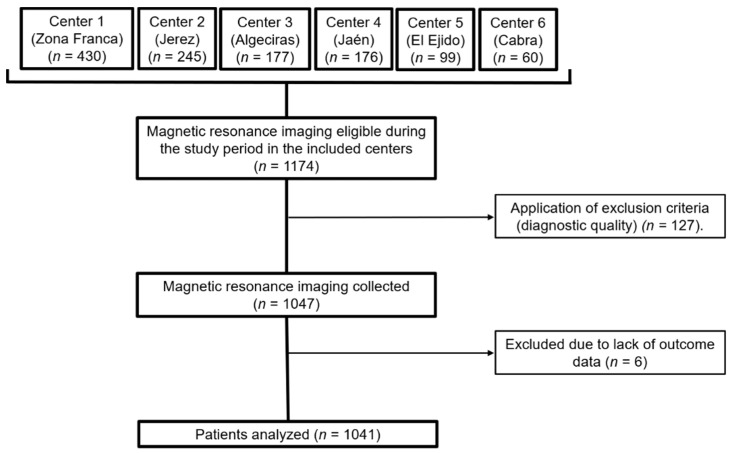
Flow diagram of the sample selection. MRI, magnetic resonance imaging.

Our sample consisted of 1041 patients who underwent brain MRI due to headache. The flow chart of the selection process is shown in Figure 1. Of the analyzed sample, a structural finding that could explain headache was found in 224 (21.5%) patients.

Table 1 shows the main characteristics of the sample, stratified by outcome (1), the presence of a structural finding that could potentially explain headache. The factors associated with this outcome in bivariate analyses were the medical specialty of the physician who ordered the MRI (higher frequency of abnormal brain structural finding when the MRI was ordered by oncologists or neurosurgeons) (*p* = 0.017), use of GBCA (*p* = 0.001), previous MRI exams (*p* = 0.024), and previously-known conditions that may justify headache (e.g., cancer) (*p* < 0.001).

Table 2 shows the stratification analyses of the exposure variable by outcomes (2) and (3) (presence of intracranial space-occupying lesions and presence of ITs, respectively). No associations were found regarding space occupying-lesions. The factors associated with the presence of IT were female sex (*p* = 0.048) and concomitant symptoms (*p* = 0.033). A detailed description of the specific concomitant symptoms is shown in Table 3. We found no associations between the specific symptoms and structural findings.

To evaluate the independent effect of these variables on outcome (1) adjusted for potential confounders, multivariate analyses were applied (Table 4). Female sex (OR = 1.35; 95% CI: 1.02–1.85), presence of concomitant symptoms (OR = 1.34; 95% CI: 1.05–1.89), and previously-known conditions potentially explaining headache (OR = 2.44; 95% CI: 1.55–3.84) were associated with the presence of structural findings in the logistic regression models. The use of GBCA was the diagnosis-related factor associated with the presence of a structural finding explaining headache (OR = 1.89; 95% CI: 1.31–2.72) in the adjusted models. The Cox and Snell R^2^ coefficient of determination of the model was 0.27.

**Figure 2:**
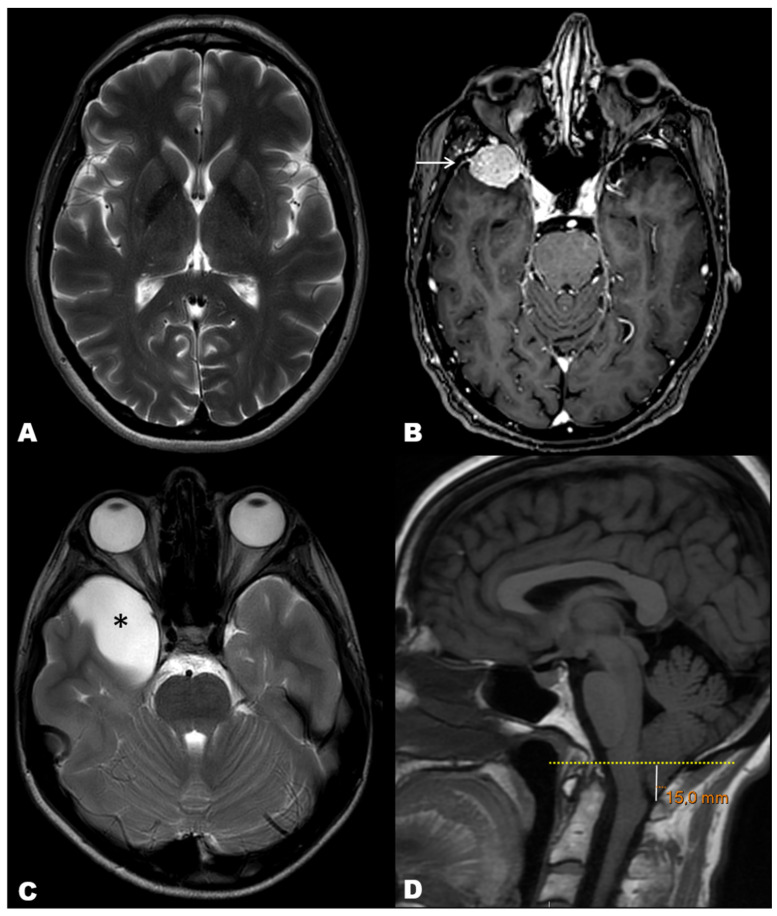
Illustrative cases of our cohort of MRI exams performed to rule out secondary causes of headache. (**A**) T2-weighted axial image in a 65-year-old woman depicting normal brain with no structural abnormalities. (**B**) Post-contrast T1-weighted axial image in a 71-year-old woman showing a right anterior temporal tumor with homogeneous enhancement and ‘dural tail’ sign, consistent with meningioma (arrow). (**C**) T2-weighted axial image in a 10 year-old boy showing a right anterior temporal cystic mass, consistent with arachnoid cyst (asterisk). (**D**) T1-weighted sagittal image in a 31 year-old man showing abnormal descent of the cerebellar amigdala below the plane of the foramen magnus, consistent with Chiari type 1 malformation.

Figure 2 illustrates examples of MRI diagnoses of each outcome of the study: normal MRI, neoplastic space-occupying lesion (meningioma), non-neoplastic space-occupying lesion (arachnoid cyst), and structural non-space-occupying lesion (e.g., Chiari I malformation). The specific findings regarding the three outcome variables of the study are summarized in Table 5.

## 4. Discussion

In this real-world clinical study, we have analyzed the prevalence of structural alterations on brain MRI that may explain the presence of headache. We found an overall prevalence of 21.5% structural alterations (6.8% SOLs and 1.5% ITs). The main factors associated with structural alterations in the adjusted models were female sex, symptoms accompanying headache, use of GBCAs and previously known conditions potentially explaining headache.

We found a clear overall predominance of women over men in our series. This may be explained by the higher prevalence of headache in the female sex [7], which may demand more medical assistance than men. Assessing the usefulness of neuroimaging studies in detecting potentially treatable structural pathologies in patients with headache has an impact on the health of the population for several reasons. First, such detection results in a direct benefit in the life of patients, especially in the case of malignant neoplasms, since delayed diagnosis usually prevents patients from being candidates to surgical management. Second, even when the structural anomalies are not treatable, being able to inform the patient adequately can help reduce their anxiety, improving their ability to cope with the disease. In addition, the negative predictive value of brain MRI should be taken into account, since demonstrating the absence of structural anomalies will likely decrease the patient’s fear of a fatal lesion and the anxiety of a worse vital prognosis, decreasing healthcare costs secondary to excessive demand of health resources [19]. Finally, in this same vein, the health care costs derived from neuroimaging studies should also be considered; in the United States, the average cost of a brain MRI exam is 660 dollars (double that of a CT scan [20]) and makes up 1 billion dollars in annual costs [21]. In European countries the cost is lower, but it still represents a substantial part of the indirect costs derived from headache, which in 2010 was estimated to be 27 million euros in the European Union [22]. In addition, knowledge of the epidemiological and clinical factors associated with a higher probability of detecting structural alterations on brain MRI is helpful for clinicians and radiologists, as it allows establishing a more appropriate workflow and triaging of patients. This is especially important in geographic areas with scarce resources. Moreover, this knowledge allows optimizing strategies from the neuroimaging point of view, for example by adapting imaging protocols (type and duration of imaging acquisitions, use of contrast agents, etc.). In addition, knowledge of the epidemiological and clinical factors associated with a higher probability of detecting structural alterations on brain MRI is helpful for clinicians and radiologists, as it allows establishing a more appropriate workflow and triaging of patients. This is especially important in geographic areas with scarce resources. Moreover, this knowledge allows optimizing strategies from the neuroimaging point of view, for example by adapting imaging protocols (type and duration of imaging acquisitions, use of contrast agents, etc.).

Regarding the overall prevalence of structural abnormalities, SOLs and ITs observed in our cohort, it is significantly higher than that reported in other studies in symptomatic patients (ranging from 0.9% to 14.1% [23,24,25,26]) including those with chronic headache (2–3%) [19,27], and in healthy subjects [28,29,30,31]. However, similar figures to ours have been reported elsewhere [32]. Discrepancies could be explained by differences in patient selection (e.g., oncologic patients were included), imaging protocols (e.g., some previous studies did not include sagittal T1 sequences, limiting the assessment of the posterior fossa) [23,33], patients’ inclusion criteria (e.g., differences in sociodemographic characteristics) [28], or the criteria used to consider a lesion as a potential cause of headache (i.e., we included lesions with mass effect or increasing intracranial pressure). In the latter regard, it should be taken into account that the way in which structural alterations are grouped according to their potential clinical association with headache may lead to important variations in prevalence [34]. For instance, some series such as that of Sempere et al. did not include sinusitis among the causes of headache [23]. In fact, headache secondary to rhinosinusitis, whether acute or chronic, is a particular subtype of special importance due to the absence of a clinically defined presentation that requires high suspicion on clinical evaluation, particularly in the case of sphenoidal sinusitis [14]. Accordingly, it represents a diagnostic challenge, and neuroimaging can be useful to determine its plausibility in patients with headache, particularly when quantification scores such as the Lund-McClay scale are used [35,36]. In our study, we included all cases in which the presence of inflammatory changes in the paranasal sinuses had been explicitly indicated in the radiology report, regardless of their extent. Consequently, we could have overestimated the causal role of paranasal inflammatory changes as a structural cause of headache. Therefore, our data in this regard should be interpreted cautiously. Future studies quantifying the degree of sinusitis-related changes on MRI should be performed to overcome this limitation.

One of the main medical reasons to order brain MRI in clinical practice in patients with headache is ruling out a brain tumor. Therefore, it is essential to identify risk factors and patient profiles that increase the likelihood of displaying this association in order to develop appropriate prevention strategies. Headache is a clinical symptom of presentation in approximately 20% of patients with brain tumor, and its prevalence during the course of the disease increases to over 50% [37]. Ravn Munkvold et al. [38] reported that headache is a common symptom in patients with ITs, especially in younger and female patients. Our findings also support the existence of a higher frequency of headache in women with ITs. It should be emphasized that the presence of headache should be regarded as a consequence of an IT, and not vice versa. In fact, previous studies disproved that headache is associated with the development of brain tumors in women [39].

On the other hand, we observed that the medical specialty of the ordering physician influenced the likelihood of finding structural abnormalities. Overall, the highest number of requests (71%) came from Neurology. This seems logical since this is the specialty most involved in the diagnosis and management of headache. In addition, neurosurgeons and oncologists were associated with a higher frequency of positive MRI findings in bivariate analyses. This is probably due to a selection bias, since patients referred from these specialists usually have a serious condition (systemic or specifically involving the CNS), which increases the a priori probability of having structural abnormalities. Of note, it has been suggested that the addition of oncologic history as a specific sub-criterion may improve sensitivity of classification of tumor headache, considering the a priori increased risk of metastasis to the brain [40,41]. In fact, multivariate analyses showed that it was the presence of previously diagnosed conditions rather than the medical specialty of the ordering physician that was associated with presence of structural anomalies.

We observed that the presence of symptoms accompanying headache was associated with a higher frequency of structural anomalies. Although the low number of tumors in our cohort precluded multivariate regression models from being applied, the overall findings are in agreement with previous studies which found that the combination of headache with specific associated symptoms increases the positive predictive value of neuroimaging exams [42]. Of note, although tumor headache was traditionally thought to display some specific clinical characteristics (e.g., worsening in the morning, being aggravated by Valsalva-like maneuvers and accompanied by nausea and/or vomiting, with a tendency to decrease later in the day) [43], the studies performed after the advent of modern neurodiagnostic techniques have pointed out that the “classic” brain tumor headache is uncommon, particularly at the time of clinical presentation [40].

The use of GBCAs has raised awareness due to reports of major adverse events such as nephrogenic systemic fibrosis [44] and, more recently, chelate deposition in the skin, brain, globus pallidus, thalamus and dentate nuclei of still unknown clinical consequences [45]. As a consequence, strict adherence to clinical guidelines on the use of GBCAs on MRI studies has been advocated worldwide. The added value of GBCAs in MRI justifies its use in different settings, as it can help to better detect and characterize brain lesions. In clinical practice, there is great heterogeneity in MRI protocols among institutions, and adequate adherence to clinical guidelines is often not met. In our cohort, the results of multivariate regression models support the idea that the use of GBCAs adds relevant value to determine secondary causes of headache in an appropriate clinical setting. This finding disagrees with previous studies reporting that contrast material did not contribute to diagnosis compared to non-contrast brain MRI [34]. Remarkably, gadolinium is useful to detect enhancing lesions (e.g., most tumors), but does not add value in other common abnormalities such as arachnoid cysts or Chiari malformation. Furthermore, it should be emphasized that the use of GBCAs in our cohort was occasionally determined by the ordering physician when explicitly asking for contrast-enhanced brain MRI, but mainly by the radiologist’s judgment based on the clinical information provided in the electronic order. Because we did not differentiate between both scenarios in our study design, it is not possible to accurately discern whether the benefits of contrast-enhanced MRI are intrinsic or dependent on the clinical skills of the ordering physician. This limitation should be overcome in future studies.

This study has several strengths. First, it is a real-world clinical study. Second, it is focused on MRI, which overcomes the limitations of other studies including CT and MRI, with homogeneous imaging protocols, blinded imaging interpretation with experienced neuroradiologists, and a multicentric nature (which overcomes limitations derived from geographic clusters of conditions such as tumors). The main limitations of this study lie in its retrospective and observational nature, which limited the type and quality of information analyzed (e.g., abnormal neurological examination), and the low sample size in two outcomes (i.e., SOLs and ITs) which precluded us from performing multivariate analyses. Another perceived limitation is that the classification of outcomes was designed according to our clinical criteria given the great heterogeneity of previously published studies on this topic. Finally, we only included as covariates in the multivariate regression analyses those variables that had been previously collected according to our a priori hypothesis; therefore, there might be other confounders that have not been considered. Given these limitations, our results should be interpreted cautiously.

Our results may be relevant for different medical specialists involved in the diagnosis, management and prevention of headache. Moreover, the risk factors identified in our study might help the development of public health strategies aimed at early diagnosis of brain tumors. Future studies are warranted to corroborate our findings.

## 5. Conclusions

In this multi-center real-world clinical study, the prevalence of structural abnormalities potentially explaining headache in brain MRI was high. Female sex and accompanying symptoms were associated with a higher frequency of structural abnormalities including intracranial tumors, in agreement with previous studies. Other factors that seem to influence the detection of structural anomalies include use of contrast material and previously known conditions potentially explaining headache such as cancer. These findings reinforce the utility of brain MRI in clinical practice and call for prospective validation of the sociodemographic and clinical profiles observed in our study to increase the impact of neuroimaging in population’s health. As a final remark, the aim of neuroimaging should be maximizing its sensitivity in detecting abnormal structural findings, even at the expense of decreasing its specificity. The requesting physician should eventually decide whether the detected lesion may explain the type of headache suffered by the patient.

## Figures and Tables

**Table 1:** Main characteristics of the sample (patients who underwent magnetic resonance imaging due to headache), stratified by the presence of a structural finding that could explain headache.

Characteristic	Total Sample	Structural Finding Explaining Headache	No Structural Finding Explaining Headache	*p*-Value ^1^
Total	1041 (100.0%)	224 (21.5%)	817 (78.5%)	-
Sex, *n* (%)				0.080
Women	728 (69.9%)	146 (20.1%)	582 (79.9%)	
Men	313 (30.1%)	78 (24.9%)	235 (75.1%)	
Age, x (sd)	38.6 (18.9)	38.6 (18.8)	38.6 (18.9)	0.980
Province, *n* (%)				0.200
Almería	88 (8.5%)	22 (25.0%)	66 (75.0%)	
Cádiz	748 (71.9%)	159 (21.3%)	589 (78.7%)	
Córdoba	53 (5.1%)	6 (11.3%)	47 (88.7%)	
Jaén	152 (14.6%)	37 (24.3%)	115 (75.7%)	
Specialty of ordering physician, *n* (%)				0.017 *
Neurology	738 (71.0%)	148 (20.1%)	590 (79.9%)	
Pediatrics	112 (10.8%)	29 (25.9%)	83 (74.1%)	
Internal Medicine	64 (6.2%)	10 (25.0%)	54 (75.0%)	
Oncology	29 (2.8%)	12 (41.4%)	17 (58.7%)	
Primary Care	24 (2.3%)	6 (25.0%)	18 (75.0%)	
Neurosurgery	22 (2.1%)	9 (40.9)	13 (59.1%)	
Others	51 (4.9%)	10 (19.6%)	41 (80.4%)	
Type of headache, *n* (%)				0.085
Migraine	225 (21.6%)	39 (17.3%)	186 (82.7%)	
Other	816 (78.4%)	185 (22.7%)	631 (77.3%)	
Contrast (Gadolinium), *n* (%)				0.001 *
Yes	176 (16.9%)	55 (31.3%)	121 (68.8%)	
No	865 (83.1%)	169 (19.5%)	696 (80.5%)	
New (incident) headache, *n* (%)				0.211
Yes	131 (12.6%)	34 (26.0%)	97 (74.0%)	
No	910 (87.4%)	190 (20.9%)	720 (79.1%)	
Concomitant symptoms, *n* (%)				0.163
Yes	304 (29.2%)	57 (18.8%)	247 (81.3%)	
No	737 (70.8%)	167 (22.7%)	570 (77.3%)	
Previous trauma, *n* (%)				0.572
Yes	27 (2.6%)	7 (25.9%)	20 (74.1%)	
No	1104 (97.4%)	217 (21.4%)	797 (78.6%)	
Previous MRI, *n* (%)				0.024 *
Yes	151 (14.5%)	43 (28.5%)	108 (71.5%)	
No	890 (85.5%)	181 (20.3%)	709 (79.7%)	
Previous known condition that may justify headache, *n* (%)				<0.001 *
Yes	106 (10.2%)	41 (38.7%)	65 (61.3%)	
No	934 (89.8%)	183 (19.6%)	751 (80.4%)	

Data are presented as absolute frequency (*n*) and relative frequency (%) for qualitative variables and as mean (x) and standard deviation (sd) for quantitative variables. ^1^ *p*-value of the *t*-test for continuous variables (age), and chi-square test for qualitative variables. When the conditions of application of the chi-square test were not met, the Fisher’s exact test was applied. * *p* < 0.05

**Table 2:** Main characteristics of the subgroup of patients who presented a structural finding potentially explaining headache (*n* = 224), stratified by the presence of space-occupying lesions (SOL) and, from this subgroup, stratified by the presence of intracranial tumors (IT).

Characteristic	Presence of SOL	Absence of SOL	*p*-Value ^1^	Presence of IT	Absence of IT	*p*-Value ^2^
Total	71 (6.8%)	970 (93.2%)	-	16 (22.5%)	55 (77.5%)	-
Sex, *n* (%)			0.718			
Women	51 (7.0%)	677 (93.0%)		13 (25.5%)	38 (74.5%)	0.048 *
Men	20 (6.4%)	293 (96.3%)		3 (15.0%)	17 (85.0%)	
Age, x (sd)	39.5 (19.3)	38.5 (18.9)	0.688	40.7 (23.4)	39.1 (18.2)	0.778
Province, *n* (%)			0.752			0.636
Almería	5 (5.7%)	83 (94.3%)		2 (25.0%)	6 (75.0%)	
Cádiz	55 (7.4%)	693 (92.6%)		12 (21.8%)	43 (78.2%)	
Córdoba	3 (5.7%)	50 (94.3%)		0 (0.0%)	3 (100.0%)	
Jaén	8 (5.3%)	144 (94.7%)		2 (25.05%)	6 (75.0%)	
Specialty of ordering physician, *n* (%)			0.493			0.241
Neurology	50 (6.8%)	688 (93.8%)		12 (24.0%)	38 (76.0%)	
Pediatrics	9 (8.0%)	103 (92.0%)		3 (33.3%)	6 (66.7%)	
Internal Medicine	5 (7.8%)	59 (92.2%)		0 (0.0%)	5 (100.0%)	
Oncology	2 (6.9%)	27 (93.1%)		1 (50.0%)	1 (50.0%)	
Primary Care	1 (4.2%)	23 (95.8%)		0 (0.0%)	1 (100.0%)	
Neurosurgery	1 (4.5%)	21 (93.2%)		0 (0.0%)	1 (100.0%)	
Others	3 (5.9%)	48 (94.1%)		0 (0.0%)	3 (100.0%)	
Type of headache, *n* (%)			0.918			0.452
Migraine	15 (6.7%)	210 (93.3%)		4 (26.7%)	11 (73.3%)	
Others	56 (6.9%)	760 (93.1%)		12 (21.4%)	44 (78.6%)	
Contrast (Gadolinium), *n* (%)			0.511			0.836
Yes	10 (5.7%)	166 (94.3%)		2 (20.0%)	8 (80.0%)	
No	61 (7.1%)	804 (92.9%)		14 (23.0%)	47 (77.0%)	
New (incident) headache, *n* (%)			0.981			0.981
Yes	9 (6.9%)	122 (93.1%)		2 (22.2%)	7 (77.8%)	
No	62 (6.8%)	848 (93.2%)		14 (22.6%)	48 (77.4%)	
Concomitant symptoms, *n* (%)			0.249			0.417
Yes	25 (8.2%)	279 (91.8%)		9 (36.0%)	16 (64.0%)	0.033 *
No	46 (6.2%)	691 (93.8%)		7 (15.2%)	39 (84.8%)	
Trauma, *n* (%)			0.439			0.225
Yes	1 (3.7%)	26 (96.3%)		1 (100.0%)	0 (0.0%)	
No	70 (6.9%)	944 (93.1%)		15 (21.4%)	55 (78.6%)	
Previous MRI, *n* (%)			0.133			0.719
Yes	6 (4.0%)	145 (96.0%)		1 (16.7%)	5 (83.3%)	
No	65 (7.3%)	825 (92.7%)		15 (23.1%)	50 (76.9%)	
Previous known condition that may justify the headache, *n* (%)			0.188			0.903
Yes	4 (3.8%)	102 (96.2%)		1 (25.0%)	3 (75.0%)	
No	67 (7.2%)	867 (92.8%)		15 (22.4%)	52 (77.6%)	

Data are presented as absolute frequency (*n*) and relative frequency (%) for qualitative variables and as mean (x) and standard deviation (sd) for quantitative variables. All statistical tests were chi-square tests except for age (*t*-test). When the conditions of application of the chi-square test were not met, the Fisher’s exact test was applied. ^1^ *p*-value of the comparison between patients that presented SOL vs. patients without SOL, of the subgroup of patients with a structural finding that could explain headache. ^2^ *p*-value of the comparison between patients with IT vs. patients without IT of the subgroup of patients with SOL. * *p* < 0.05.

**Table 3:** Concomitant symptoms reported by the patients who required magnetic resonance imaging due to headache.

Concomitant Symptoms	*n* (%) from the Total Sample (*n* = 1041)	% from the Patients with Concomitant Symptoms (*n* = 100)	*
None	737 (70.8)	-	0.184
≥1 symptom	304 (29.2)	-	
Visual disturbances	89 (8.9)	29.3	0.594
Vertigo or dizziness	58 (5.6)	19.1	0.324
Paresthesia	58 (5.6)	19.1	0.407
Instability	28 (2.7)	9.2	0.372
Non-cardiogenic syncope	18 (1.7)	5.9	-
Disorientation or memory disturbance	17 (1.6)	5.6	-
Motor impairment or movement disorders	13 (1.3)	4.3	-
Tinnitus	12 (1.1)	3.9	-
Epileptic seizures	10 (1.0)	3.3	-
Hypoacusis	1 (0.1)	0.3	-

* *p*-value of the association between each symptom and the presence of structural abnormalities. Chi-square tests and Fisher exact tests were applied as appropriate. For low-frequent symptoms (*n* < 20), no bivariate analyses were conducted.

**Table 4:** Multivariate logistic regression models for the diagnosis of structural findings explaining headache on magnetic resonance imaging (MRI).

Variables	Crude OR (95% CI)	Adjusted OR ^1^ (95% CI)
Sex (female)	1.32 (0.97–1.81)	1.35 (1.02–1.85) *
Age	1.00 (0.99–1.01)	0.99 (0.98–1.01)
Type of headache (non-migraine)	1.40 (0.95–2.05)	1.27 (0.86–1.88)
Use of contrast (gadolinium)	1.88 (1.31–2.68)	1.89 (1.31–2.72) *
Presence of accompanying symptoms	1.27 (0.91–1.78)	1.34 (1.05–1.89) *
Previous MRI	1.56 (1.06–2.30)	1.25 (0.82–1.90)
Previously known condition potentially explaining headache	2.59 (1.70–3.95)	2.44 (1.55–3.84) *

^1^ Odds ratios (ORs) adjusted for the variables included in the table. The reference groups were the opposite of those shown in brackets (i.e., sex (male), migraine, non-use of gadolinium, absence of concomitant symptoms, absence of previous MRI exams, and absence of previously known condition potentially explaining headache). * Significant OR in the adjusted model (the null value is not included in the 95% CI).

**Table 5:** Frequency of specific structural abnormalities in the sample.

Structural Abnormalities	*n* (%)
**Total**	224 (100.0)
**Non-space-occupying lesions**	153 (68.3)
Sinusitis	119 (53.1)
Chiari malformation	10 (4.5)
Non-communicating hydrocephalus	8 (3.6)
Pseudotumor cerebri	7 (3.1)
Dural sinus thrombosis	5 (2.2)
Arteriovenous malformation	2 (0.9)
Subacute stroke	2 (0.9)
**Space-occupying lesions**	71 (31.7)
Arachnoid cyst	32 (14.3)
Subdural hygroma	8 (3.6)
Aneurism	4 (1.8)
Cavernoma	4 (1.8)
Epidermoid cyst	3 (1.3)
Chronic subdural hematoma	2 (0.9)
Intraparenchymal hematoma	1 (0.4)
Subdural empyema	1 (0.4)
Intracranial tumors	16 (7.1)
Meningioma	7 (3.1)
Metastasis	7 (3.1)
Glioma	2 (0.9)

## Data Availability

The data used in this study are available upon reasonable request to the corresponding author.
